# Evaluation of the protective efficacy of Olyset®Plus ceiling net on reducing malaria prevalence in children in Lake Victoria Basin, Kenya: study protocol for a cluster-randomized controlled trial

**DOI:** 10.1186/s13063-023-07372-3

**Published:** 2023-05-25

**Authors:** Wataru Kagaya, Chim W. Chan, James Kongere, Bernard N. Kanoi, Mtakai Ngara, Protus Omondi, Ashley Osborne, Laura Barbieri, Achyut Kc, Noboru Minakawa, Jesse Gitaka, Akira Kaneko

**Affiliations:** 1 Department of Virology and Parasitology/Research Center for Infectious Diseases, Graduate School of Medicine, Osaka Metropolitan University, Osaka, Japan; 2 Directorate of Research and Innovation, Mount Kenya University, Thika, Kenya; 3 Department of Microbiology, Tumor and Cell Biology, Karolinska Institutet, Stockholm, Sweden; 4 Faculty of Infectious and Tropical Diseases, London School of Hygiene and Tropical Medicine, London, UK; 5 Department of Vector Ecology and Environment, Institute of Tropical Medicine, Nagasaki University, Nagasaki, Japan

**Keywords:** Malaria, *Anopheles* mosquito, Vector control, LLIN, Pyrethroid resistance, Ceiling net, Kenya, Cluster-randomized controlled trial

## Abstract

**Background:**

In the Lake Victoria Basin of western Kenya, malaria remains highly endemic despite high coverage of interventions such as insecticide-impregnated long-lasting insecticidal nets (LLIN). The malaria-protective effect of LLINs is hampered by insecticide resistance in *Anopheles* vectors and its repurposing by the community. Ceiling nets and LLIN with synergist piperonyl butoxide (PBO-LLIN) are novel tools that can overcome the problems of behavioral variation of net use and metabolic resistance to insecticide, respectively. The two have been shown to reduce malaria prevalence when used independently. Integration of these two tools (i.e., ceiling nets made with PBO-LLIN or Olyset®Plus ceiling nets) appears promising in further reducing the malaria burden.

**Methods:**

A cluster-randomized controlled trial is designed to assess the effect of Olyset®Plus ceiling nets on reducing malaria prevalence in children on Mfangano Island in Homa Bay County, where malaria transmission is moderate. Olyset®Plus ceiling nets will be installed in 1315 residential structures. Malaria parasitological, entomological, and serological indicators will be measured for 12 months to compare the effectiveness of this new intervention against conventional LLIN in the control arm.

**Discussion:**

Wider adoption of Olyset®Plus ceiling nets to complement existing interventions may benefit other malaria-endemic counties and be incorporated as part of Kenya’s national malaria elimination strategy.

**Trial registration:**

UMIN Clinical Trials Registry UMIN000045079. Registered on 4 August 2021.

## Administrative information

**Table:** 

Title {1}	Evaluation of the protective efficacy of Olyset®Plus ceiling net on reducing malaria prevalence in children in Lake Victoria Basin, Kenya: study protocol for a cluster-randomized controlled trial
Trial registration {2a and 2b}	University Hospital Medical Information Network (UMIN) Clinical Trials Registry, Japan, UMIN000045079, registered on 4th August 2021
Protocol version {3}	Version 2.0 (1st February 2022)
Funding {4}	This work is supported by the Japan International Cooperation Agency (JICA) and Japan Agency for Medical Research and Development (AMED) under the Science and Technology Research Partnership for Sustainable Development Goals (SATREPS) program.
Author details {5a}	Wataru Kagaya^1,2^, Chim W. Chan^1^, James Kongere^1^, Bernard N. Kanoi^2^, Mtakai Ngara^2,3^, Protus Omondi^1^, Ashley Osborne^4^, Laura Barbieri^1^, Achyut Kc^1, 3^, Noboru Minakawa^5^, Jesse Gitaka^2^, Akira Kaneko^1,3,5^ 1. Department of Virology and Parasitology/Research Center for Infectious Diseases, Graduate school of Medicine, Osaka Metropolitan University, Osaka, Japan 2. Directorate of Research and Innovation, Mount Kenya University, Thika, Kenya 3. Department of Microbiology, Tumor and Cell Biology, Karolinska Institutet, Stockholm, Sweden 4. Faculty of Infectious and Tropical Diseases, London School of Hygiene and Tropical Medicine, London, UK 5. Department of Vector Ecology and Environment, Institute of Tropical Medicine, Nagasaki University, Japan
Name and contact information for the trial sponsor {5b}	Department of Virology and Parasitology/Research Center for Infectious Diseases, Graduate School of Medicine, Osaka Metropolitan University (OMU), Japan 1–4-3, Asahimachi, Abeno, Osaka, Osaka, Japan, 545–8585 TEL: + 81–6-6645–3760 Website: https://ocuparasitology.com/en/ Directorate of Research and Innovation, Mount Kenya University (MKU), Kenya General Kago Road, Thika, Kiambu, Kenya Website: https://www.mku.ac.ke
Role of sponsor {5c}	OMU will support project management oversight, trial management, data management, statistical analysis, and research governance. MKU also holds overall authority together with project management and analysis.

## Introduction

### Background and rationale {6a}

Vector control is a key component of malaria control programs worldwide. Among several vector control strategies, bed nets are the most widely adopted tool to prevent mosquito bites and malaria transmission. Insecticide-treated nets (ITN), especially long-lasting insecticidal nets (LLIN), are the single most important contributor to the remarkable reduction of malaria cases and deaths since the early 2000s [1, 2]. However, that trend has stalled since 2015. Malaria cases are even increasing in some countries [3], suggesting the need to optimize the use of existing tools, including LLIN, malaria rapid diagnostic tests (RDT), and artemisinin-based combination therapy (ACT), as well as the addition of novel tools and an update of current control combination strategies.

Repurposing and inconsistent uses of LLIN are widely observed where mass LLIN distribution has been implemented. These may act as key factors in reducing the impact of LLIN. For example, in the Lake Victoria region of Kenya, the repurposing of LLIN for fishing and protecting crops and livestock are well known [4, 5]. Sharing a single LLIN by more than two persons can also reduce its protective effect [6]. Furthermore, our previous study showed that schoolchildren, who are most vulnerable to malaria infection, tend to sleep in the living room without bed nets [7].

Additionally, several studies have demonstrated that house screening reduces the number of mosquitoes entering the structure and also protects those who do not use bed nets properly [6, 8]. Screens that cover the ceiling and the gap between the ceiling and the walls (ceiling nets) are especially effective since these gaps are the major entry points for mosquitoes into the house [9, 10], and mosquitoes habitually rest on walls or ceilings after a blood meal [11, 12]. Hence, ceiling nets with insecticide-treated material can kill infected mosquitoes and interrupt malaria transmission [13, 14]. Moreover, once installed, ceiling nets demand little to no input from users, unlike conventional bed nets that require setting before sleep and removing in the morning; hence, the impact may be less influenced by inconsistent human behaviors [15–17].

Resistance to pyrethroid insecticides in mosquitoes can undermine the current LLIN strategy. There are currently two main mechanisms of insecticide resistance in anopheline mosquitoes, target-site mutations in the pyrethroid receptor and metabolic resistance [18]. Metabolic resistance is caused by allelic and expression changes in cytochrome P450 enzymes that detoxify pyrethroids [19]. To combat this type of pyrethroid resistance, Olyset®Plus, an LLIN incorporating the insecticide synergist piperonyl butoxide (PBO), has been developed. PBO inhibits the enzymes that break down pyrethroids. Previous studies have demonstrated the increased insecticidal effect of Olyset®Plus when compared with the standard LLINs, even in areas where pyrethroid-resistant mosquitoes were widely reported [20–22].

Integration of ceiling nets and LLIN with PBO (Olyset®Plus ceiling nets) can address the major shortcomings of current LLIN and provide additional protection against malaria over individual adoption of either tool. However, a few malaria-endemic areas, including our study area, still have high malaria prevalence even after the wide and repeated distribution of LLIN to the community, suggesting that the current LLIN program is insufficient for combating residual malaria transmission. Olyset®Plus ceiling net has the potential as one of the updates of the current LLIN. Here, we designed a cluster randomized controlled trial to evaluate its protective efficacy in reducing malaria prevalence. The most vulnerable population to malaria is small children. However, young adults or adults are also an important population in malaria transmission since they can act as a reservoir of transmission as asymptomatic infections. Thus, the proposed study includes monitoring two target populations: school-age children (3 to 15 years old) and all age groups.

### Objectives {7}

The primary study objective is to determine the protective efficacy of Olyset®Plus ceiling nets in reducing malaria prevalence in children aged 3 to 15 years old, during a 12-month follow-up period.

The secondary objectives during a 12-month follow-up period are as follows: (1) to determine the total number of infections averted due to Olyset®Plus ceiling net by comparing the cumulative number of infections per person per year in cohort participants in the intervention and control arms; (2) to determine the protective efficacy of Olyset®Plus ceiling net in reducing anemia prevalence in children aged 3 to 15 years old; (3) to measure the impact of Olyset®Plus ceiling net on entomological parameters (anopheline mosquito density, composition, and malaria infection rate), (4) to measure the impact of the introduction of Olyset®Plus ceiling net on malaria preventive behavior, especially bed net usage after the 12 months of the intervention; and (5) to assess the acceptance of Olyset®Plus ceiling net within the community.

### Trial design {8}

The study is a cluster-randomized controlled trial with 10 clusters per arm (intervention and control), consisting of two cross-sectional school surveys after 6 months and 12 months of intervention and one cohort followed for 12 months in both arms. Each cluster is a health unit comprising approximately 100 households, including 500 individuals. Each health unit has an assigned community health volunteer (CHV) responsible for national health surveys and routine community health activities. This study also utilizes CHVs as messengers of study-related information and enumerators of the questionnaire surveys.

To evaluate the primary outcome, 150 schoolchildren will be recruited from each cluster. To evaluate the protective efficacy in reducing the cumulative incidence, 25 individuals will be recruited from each cluster and followed up for 12 months as a cohort. Residents of all age groups will be recruited to allow for the evaluation of the impact on asymptomatic and/or submicroscopic infections, which are also observed in adults. Cohort participants will be interviewed about their health conditions every 2 weeks by CHVs and are asked to provide a blood sample to monitor asymptomatic infection. The blood collection will be done by finger prick and venipuncture alternately. Five house structures in each cluster will be randomly selected and subjected to pyrethrum spray catches (PSC) for the entomological assessment. The mosquito sampling will be implemented 6 and 12 months after the intervention. Sampling will take place at the same structures at both time points.

## Methods: participants, interventions, and outcomes

### Study setting {9}

**Fig. 1:**
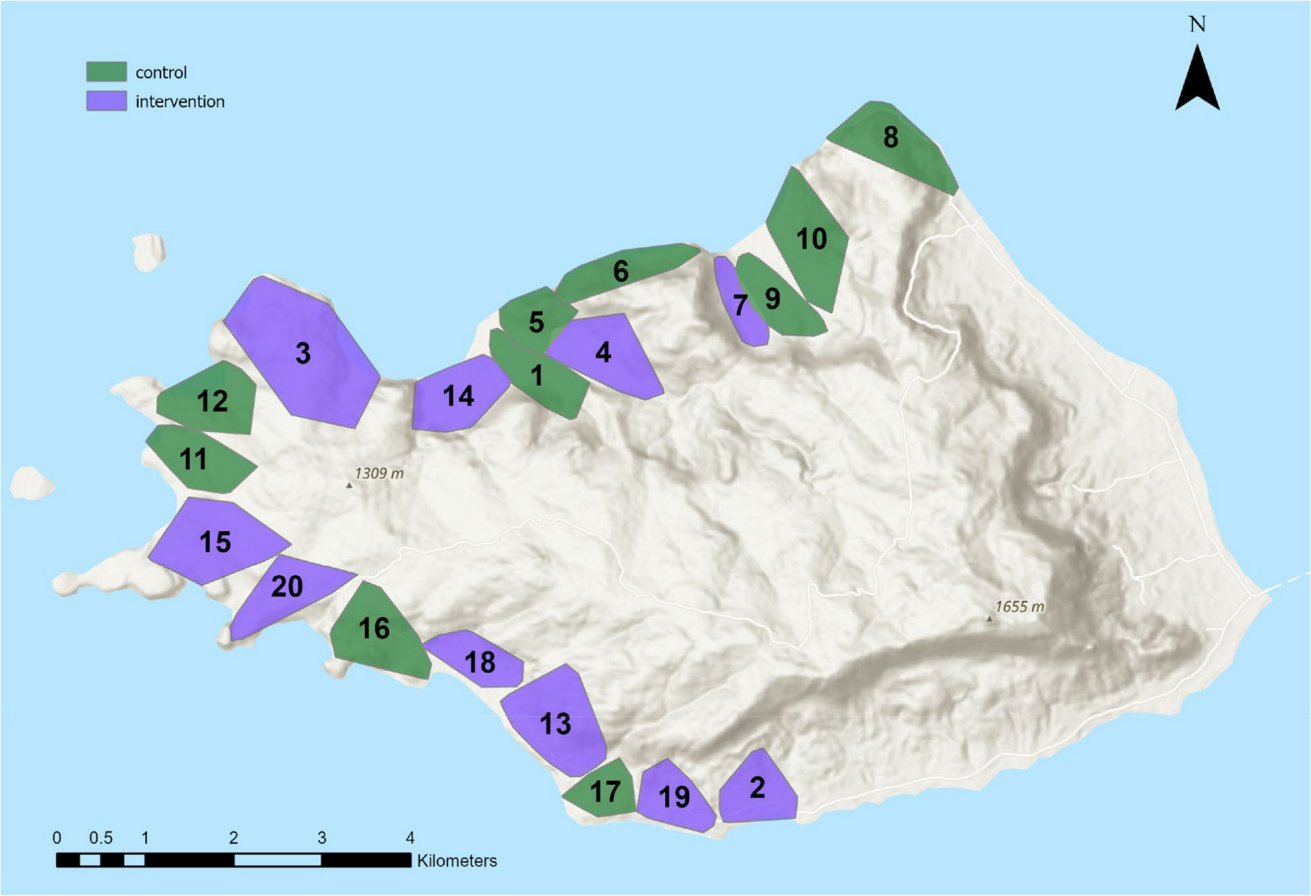
Map of Mfangano Island showing the locations of the 20 trial clusters

The study will be conducted on Mfangano Island in Homa Bay County, Kenya (Fig. 1). According to the most recent national census in 2019, the island has a land area of 66.2 km^2^ and a population of 24,123 distributed in 6085 households [23]. Luo and Suba are the major and minor ethnic groups on the island, respectively, with significant admixture between the two groups. The primary occupations of Mfangano residents include fishing in Lake Victoria and farming. Most compounds possess more than two house structures, and family dwell in each structure is referred to as a household. The main dwelling units are typically made of mud walls with metal sheet roofs, although units with walls made of iron sheets, concrete, or stones are also common [23].

In general, the Lake Victoria region has two rainy periods per year, including the long rainy season from March to June and the short rainy season from October to November. However, irregular patterns have been observed in the last few years [24]. Malaria incidence peaks 1 to 2 months after the rainy season. The major vectors are *Anopheles gambiae* s.s., *An. arabiensis*, and *An. funestus* [25].

Mfangano has six public health facilities (three health centers and three dispensaries). The island is divided into 52 health units, each comprising 50 to 100 households. A CHV will monitor the health status of residents in each health unit. Under Homa Bay County government mandate, CHVs are to test suspected malaria cases by RDT and provide ACT treatments to confirmed positive cases. LLINs are distributed for free by the government every 3 years, and the most recent distribution in Homa Bay County began in March 2021.

### Eligibility criteria {10}

The inclusion criteria for the installation of the Olyset®Plus ceiling net are (1) residential structures housing at least one permanent resident aged 18 years or older in the household and (2) informed consent provided by at least one adult in the household. The exclusion criteria are (1) vacant dwelling structure (confirmed by at least two visits by CHVs) and (2) dwelling structure to be vacated or destroyed within the study period.

The inclusion criteria for cross-sectional malaria and anemia prevalence surveys in schools are (1) age between 3 and 15 years old, (2) living in the study area during the study period, and (3) informed consent being provided by the parent or legal guardian before each survey. The exclusion criterion is (1) having severe chronic illnesses.

The inclusion criteria for prospective cohorts are (1) living in the study area at the time of Olyset®Plus ceiling net installation, (2) having no plan to leave or stay outside the study area for an extended period (longer than 1 month) over the 12-month follow-up period, and (3) informed consent provided (if the participant is minor (less than 18 years old), informed consent has to be provided by the participant’s parent or guardian). The exclusion criteria are (1) severe chronic illnesses and (2) pregnancy at the time of Olyset®Plus ceiling net installation. Table 1 summarizes these criteria.

**Table 1:** The inclusion and exclusion criteria for ceiling net installation, cross-sectional survey, and prospective cohort survey

Inclusion criteria	Exclusion criteria
**Ceiling net installation**	
At least one permanent resident aged 18 years or older in the household	Vacant dwelling structure (confirmed with at least two visits by CHVs)
Informed consent provided by at least one adult in the household	Dwelling structure to be vacated or destroyed within the study period
**Cross-sectional survey**	
Age between three and 15 years old	Severe chronic illnesses
Living in the study area during the study period	
Informed consent was provided by the parent or guardian before each survey	
**Prospective cohort survey**	
Living in the study area at the time of Olyset®Plus ceiling net installation	Severe chronic illnesses
No plan to leave or stay outside the study area for an extended period (longer than one month) over the 12-month follow-up period	Pregnancy
Informed consent provided (if the participant is a minor, informed consent must be provided by the participant’s parent or guardian)	

### Who will take informed consent? {26a}

Written informed consent will be obtained by the study team members fluent in the local languages (Luo), Kiswahili, and English and who fully understand the study protocol. After eligibility is confirmed, the study team members will present to the potential participant a document containing all relevant information about the study in Luo and English. If the participant cannot read, study information will be conveyed verbally by the study team members. The potential participant will have opportunities to ask any questions. Agreement to participate is sought only after the participant indicates an appropriate understanding of the study.

### Additional consent provisions for collection and use of participant data and biological specimens {26b}

The study information document for ceiling net installation contains the study overview. In addition, the documents for cross-sectional and cohort surveys contain details on collecting, storing, and using personal data and biological specimens during the study.

### Interventions

#### Explanation for the choice of comparators {6b}

In Kenya, LLIN is the most widely used malaria preventive measure. The Division of National Malaria Programme coordinates free LLIN distribution, and the county governments deliver LLINs to residents in all endemic counties every 3 years. The primary purpose of this trial is to demonstrate the superiority in malaria prevention of adding Olyset®Plus ceiling nets to the standard LLIN. Thus, in the control arm, no Olyset®Plus ceiling nets will be installed. Free LLIN distribution and use will be allowed in the control and intervention arms as the current best practice. As for the planning stage, there is no plan for new LLIN distribution during the study period.

#### Intervention description {11a}

In the intervention arm, Olyset®Plus ceiling nets will be installed in all dwelling units where residents sleep, free of charge to the households. All participants will be encouraged to continue to use LLINs, distributed by the Homa Bay County government.

Ceiling net installation teams consisting of experienced installers who have participated in the previous ceiling net trial [14], CHVs, and community volunteers from each health unit will schedule the installation time for each eligible household. The head (or another adult) of the eligible household will be informed at least 24 h prior to the scheduled installation time.

**Fig. 2:**
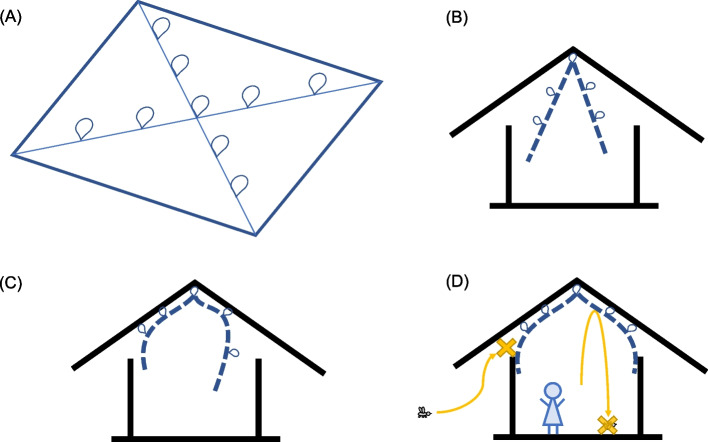
Scheme of the ceiling net and the process for the installation. **A** The rectangular LLIN sheet is reinforced along the edges and diagonals. Four loops sewn on each diagonal and one central loop sewn at the intersection of the diagonals enable the net to be tied with strings to the roof support beams and the center of the roof, respectively. **B** The central loop is tied to the center of the roof. **C** The net is pulled tight to avoid slack, and the rest of the loops are tied to the support beans. **D** The edges of the net are pinned to the top of the walls, and the gap between the walls and the roof is screened

The ceiling net material is a rectangular sheet of Olyset®Plus net measuring 6 m × 8 m or 4 m × 6 m, depending on the size of the house [8]. The ceiling net has loops along the diagonals, allowing the net to be roped to the roof support beams. After tying the central loop to the center of the roof, all four sides of the net will be pulled taut, and the remaining loops will be tied to the roof support beams. The edges of the ceiling net are then pinned to the walls, screening the opening between the roof and the walls (Fig. 2).

#### Criteria for discontinuing or modifying allocated interventions {11b}

Since the ceiling net is semi-permanently installed, the intervention will be discontinued only when the participant specifically requests the removal of the ceiling net by the study team. We do not allow any crossover from the control arm to the intervention arm during the follow-up period. Those who migrate between the arms or emigrate from the study areas will be dropped from the study follow-up.

#### Strategies to improve adherence to interventions {11c}

Adherence to the intervention cohort in this study is defined as sleeping in houses with Olyset®Plus ceiling nets. Adherence is monitored indirectly by assessing the number of nights each participant spends outside their house during the bi-weekly interview. During each house visit, CHVs will visually inspect the condition of the ceiling nets. Any visible tear and damage to the ceiling net will be reported to the research team by the size and location of the damage.

#### Relevant concomitant care permitted or prohibited during the trial {11d}

There is no specific concomitant care prohibited during the trial. All participants in both arms will continue to receive and use free LLIN and have access to standard medical care, including malaria testing by RDT and treatment with ACT.

#### Provisions for post-trial care {30}

All participants will be under the normal healthcare system in the study setting. No perceived health risks for the intended population are expected with the intervention. Our plan of continuous cross-sectional malaria surveillance after the study period allows us to monitor further parasite transmission in the population.

### Outcomes {12}

The primary outcome of the study is malaria prevalence by microscopy in schoolchildren (3 to 15 years old) 12 months after Olyset®Plus ceiling net installation. The secondary outcomes are (1) malaria prevalence by PCR in schoolchildren at 6 and 12 months post-installation; (2) cumulative malaria incidence in the prospective cohort for 12 months post-installation; (3) anemia prevalence in schoolchildren at 6 and 12 months post-installation; (4) prevalence and level of antibodies (IgG) against *Anopheles* salivary proteins gSG6 and cE5 in the prospective cohort at 6 and 12 months post-installation, as a measure of exposure to mosquito bites; (5) anopheline mosquito density, species composition, and sporozoite infection prevalence at 6 and 12 months post-installation; (6) the percentage of cohort participants who sleep under the bed net at 6 and 12 months post-installation; and (7) perception of the Olyset®Plus ceiling nets among community members.

#### Participant timeline {13}

The study flowchart and sampling timeline are presented in Table 2.

**Table 2:** The study flowchart

		Study period																
Time point (weeks)		− 8	0	2	4	6	8	10	12	14	16	18	20	22	24	…	48	50
Enrollment	Eligibility screen	X																
	Informed consent	X																
	Allocation		X															
Interventions	Install the ceiling net		X															
Assessments (cohort)	Questionnaire survey by CHVs		X	X	X	X	X	X	X	X	X	X	X	X	X	…	X	
	Venous blood sampling by a lab tech				X				X				X			…		
	Capillary blood sampling by a lab tech		X				X				X				X	…	X	
	Passive case detection			X	X	X	X	X	X	X	X	X	X	X	X	…	X	
	Qualitative survey																	X
Assessments (cross-sectional)	School malaria survey		X														X	

#### Sample size {14}

Parasite prevalence by microscopy among schoolchildren on Mfangano Island is approximately 20%, based on our previous study [26]. To detect the expected effect of 60% parasite prevalence reduction (to a prevalence of 8%) relative to control, with a power of 0.8, two-sided type 1 error of 5%, an intraclass correlation coefficient (ICC) for clusters of 0.063 (estimated based on our previous school study by assuming each school as a cluster), and average cluster size 150 (number of schoolchildren), the required number of clusters is 10 (or 1500 children) per arm using Stata/MP16.1. Based on the 2019 national census, children 3 to 15 years old account for 40.3% of the total population in Homa Bay County. The study requires a total population (all ages) of 7444 people or 1880 households (mean of 3.96 persons per household on Mfangano). By assuming a 3% dropout per month, we plan to enroll 1315 households for the intervention arm.

For the longitudinal cohort, we estimate the infection incidence on Mfangano Island as approximately 2.0 per person-year based on the data on Kenya Health Information System (KHIS). To detect the expected effect of a 40% reduction (1.2 per person-year), with a power of 0.8, two-side type 1 error of 5%, and an average cluster size of 25 individuals, the required number of clusters is 7 (or 350 individuals) per arm based on Eq. 7.3 on reference [27]. Assuming 3% of cohort participants will drop out per month, we plan to enroll 490 individuals in the cohort.

#### Recruitment {15}

##### Community sensitization

We first sought approval from the Homa Bay County Government Ministry of Health and based on their advice, hosted meetings with CHVs, village chiefs, and public health officers on Mfangano Island, and other key stakeholders from the county to explain the purpose, scope, objectives, methods, timeline, and potential significance of our study. CHVs and village leaders were asked to disseminate study information to and answer questions from community members. Feedback from CHVs and village leaders was sought, and regular meetings were held among CHVs, village leaders, and the study team to devise and refine approaches to engage communities. Finally, broad-level community consent to participate in the ceiling net trial was sought through CHVs and village chiefs.

##### Community census

After community sensitization and affirmation of agreement to participate provided by village chiefs, a census will be conducted by CHVs and experienced local research assistants to enumerate and record demographic information from all households in the health units that are receptive to our study. The following information will be collected from each household: (1) the number of residents; (2) the age, gender, and occupation of each resident; (3) the name of the school attended by each child; (4) the number, type, size, and functions of house structures; (5) the current LLIN ownership and usage; and (6) the GPS coordinates of the household. Written informed consent to participate in the ceiling net trial will be sought from the head of each household during census house visits.

### Assignment of interventions: allocation

#### Sequence generation {16a}

Random numbers are generated using Microsoft Excel 2016.

#### Concealment mechanism {16b}

Each cluster is assigned a computer-generated random number. The random numbers are then arranged in ascending order. Clusters with random numbers in the smaller half are assigned to the intervention arm, while those in the larger half are in the control arm.

#### Implementation {16c}

The allocation sequence and assignment are generated by a volunteer who has no knowledge about the study. Local study assistants will perform participant enrollment.

### Assignment of interventions: blinding

#### Who will be blinded {17a}

Due to the visibility of the Olyset®Plus ceiling net, neither the trial participants nor the members of the study team who take part in field activities can be blinded. However, laboratory- and office-based personnel (e.g., microscopists, laboratory technicians, and data analysts) will be blinded to the identity and intervention status of the trial participants since all biological specimens will be identified by a unique numeric study identifier, and personal information will be removed before analyses.

#### Procedure for unblinding if needed {17b}

This is an open-label trial, and only the data measurers are blinded. Therefore, there is no circumstance that they need to be unblinded.

### Data collection and management

#### Plans for assessment and collection of outcomes {18a}

##### Cross-sectional school surveys

Malaria prevalence in children will be estimated using cross-sectional malariometric surveys in schools. These surveys will be conducted at baseline (before ceiling net installation), 6 months, and 12 months post-installation. In addition, class rosters will be cross-referenced with the community census to determine the allocation of each eligible student in the trial clusters.

Malaria status will be determined using three methods: RDT, microscopy, and PCR. First, a finger prick blood sample will be collected for on-site diagnosis by Paracheck-Pf® RDT (Orchid Biomedical Systems, India). Survey participants with positive test results will receive a treatment course of artemether-lumefantrine with dosing instructions as per the guidelines from the Ministry of Health in Kenya. Blood smears will be prepared on-site and transported to the main laboratory in Homa Bay, where thin smears are fixed with methanol. All smears are stained with 3% Giemsa solution for 30 min, then examined by experienced microscopists. Two blood samples (70 µl each) will be collected with a 75-mm EDTA-coated micro-hematocrit capillary tube (Marienfeld, Lauda-Königshofen, Germany) and spotted on Whatman ET31 Chr filter paper (Whatman International. Maidstone, UK). The blood spots will be allowed to dry at ambient temperature and stored in individual zipped plastic bags at − 20 °C. The dried blood spots (DBS) will be used for DNA extraction and determination of malaria status by PCR [28].

Hemoglobin (Hb) levels will be measured using the HemoCue Hb 801 Analyzer (HemoCue, Ängelholm, Sweden). Survey participants with severe anemia based on the WHO criteria [29] will be referred to local health facilities for further consultation.

##### Cohort survey

Malaria incidence, exposure to *Anopheles* bites, will be assessed during and after 12 months of Olyset®Plus ceiling net installation. Every 2 weeks, CHVs will visit the homes of cohort participants. A structured questionnaire created using the Research Electronic Data Capture (REDCap) application and loaded on an Android-based tablet computer will be used to collect any history of fever, malaria episode, visit to the local health facilities, and travel in the previous 2 weeks [30].

Every month, certified medical laboratory staff will accompany CHVs on home visits to collect blood samples from cohort participants. Blood sampling by finger prick and venipuncture will alternate between monthly visits, starting with a finger prick at baseline. Blood samples will be used to determine malaria infection status by RDT, microscopy, PCR, and Hb measurement as described for the cross-sectional surveys. In addition, whole blood (500 µl for capillary blood and 3 ml for venous blood) will be collected in EDTA tubes. These samples will be transported to the main laboratory in Homa Bay County Teaching and Referral Hospital and stored at − 80 °C.

Training sessions will be held for CHVs to familiarize themselves with the questionnaire’s content and the REDCap application’s use to record the responses. Built-in validation and completion checks will ensure data quality and completeness, respectively. To avoid duplication, all microscope slides, filter papers, and sample tubes will be pre-labeled with auto-generated serial numbers. CHVs and certified medical laboratory staff will be prompted by the REDCap application before blood sampling to confirm the identity and serial number of the cohort participant. The completeness of blood sampling will be confirmed twice after the sampling step in the field and at the sample storage step in the laboratory.

##### Mosquito density, species composition, and infection rates

A cross-sectional entomological survey will be conducted to obtain baseline data before the intervention. Indoor-resting female anopheline mosquitoes will be sampled from five houses in each cluster using pyrethroid spray catches and light traps. All selected houses are made of mud walls and consist of one room. Using the baseline data, the cluster size (the number of houses) will be estimated for a post-intervention survey with a 50% density reduction and power of 0.8. The post-intervention survey will be conducted at the end of the rainy season during the study period. Sampled anophelines will be identified at the species level with PCR. The occurrence of *Plasmodium* parasites in the salivary glands of individual mosquitoes will also be examined using PCR.

##### Acceptability of Olyset®Plus ceiling nets

Focus group discussions (FGDs), a structured questionnaire, and in-depth interviews will be used to assess the acceptance of the Olyset®Plus ceiling nets. At the end of the 12-month follow-up, all cohort members will be subjected to the structured questionnaire, and a part of them will be invited to in-depth interviews based on their responses to the questionnaires.

#### Plans to promote participant retention and complete follow-up {18b}

To promote retention among participants in the control arm, we will install ceiling nets in their houses after the 12-month follow-up period, irrespective of the study outcome. This arrangement was agreed upon by CHVs and village leaders as an acceptable study incentive, conveyed to potential study participants during community sensitization, and reiterated to participants during the census survey and informed consent process.

In the cohort survey, CHVs will make an appointment with participants and confirm their available date and time before each bi-weekly home visit. Participants will receive a small remuneration (i.e., rice, beans, cooking oil, and soap) when they provide a venous blood sample. CHVs will be instructed to relay to the research team any issue raised by cohort participants, and discussions will be held to resolve issues that cannot be immediately addressed. The research team will periodically accompany the CHVs in their home visits to reinforce to cohort participants the importance of the study.

#### Data management {19}

Study data will be collected on Android-based tablet computers using the REDCap application to promote data quality and security. Data validation, such as range checks and completeness checks, will be enabled in all survey instruments. For cross-sectional surveys, data will be uploaded to the REDCap server at Mount Kenya University at the conclusion of each survey day. After the data manager confirms the data quality on the server, data stored locally on the tablet computers will be deleted before the next survey to avoid potential overwriting of existing data. Survey data will be uploaded to the REDCap server at least once a week for the longitudinal cohorts. Each cohort participant is given a unique identifier, and each visit is preprogrammed as a defined event in the REDCap data collection instrument to facilitate data entry. Cohort surveys will be conducted by CHVs familiar with cohort participants and will be prompted to confirm the identity of the cohort participants before data entry. In addition, the data manager will confirm the data quality on the server once a week.

Access to survey data will be limited to data analysts and the data manager in the research team. In addition, personally identifiable information will be removed before data analyses.

#### Confidentiality {27}

To maintain confidentiality, each participant in cross-sectional surveys and the longitudinal cohort is assigned a unique identifier. The data collected will be labeled using the unique identifier and stored separately from the key linking personal information (name, date of birth, GPS, and phone number). The data will be kept on a secure server that is only accessible to the research staff. Publications will contain only aggregated data, and no personal information will be included.

#### Plans for collection, laboratory evaluation, and storage of biological specimens for genetic or molecular analysis in this trial/future use {33}

Blood samples will be collected to examine malaria infections by multiple methods, hemoglobin levels, immunity against malaria parasites and mosquito saliva, and malaria parasite genomics. No human genetic studies are planned in this study. However, any biological specimens remaining after analyses described in this study will be stored indefinitely for future studies unless the participants opt out during the informed consent process. Contact information of the study team is provided in the consent form to study participants, who can remove themselves from this study and any future studies that may use their blood samples at any time without penalty or prejudice.

## Statistical methods

### Statistical methods for primary and secondary outcomes {20a}

The intention-to-treat (ITT) analysis is the primary analysis approach for both the primary and secondary outcomes, and the per-protocol analysis will be included as a supplementary analysis.

The primary outcome of the study is malaria prevalence by microscopy in schoolchildren (3 to 15 years old) 12 months after Olyset®Plus ceiling net installation. The results of microscopic examination from the cross-sectional survey 12 months after the implementation will be compared between the intervention arm and the control arm, based on cluster-level summaries using the two-stage procedure [27]. In the first stage, an individual-level logistic regression model will be constructed with adjustments for confounders, including age, gender, bed net use, house structure, and SES, and a fitting value will be summarized for each cluster using the model. In the second stage, a residual from the fitted values and the observed values for each cluster will be computed. The difference between the two groups will be tested using Wilcoxon’s rank-sum test by ranking cluster-level summaries.

As a secondary outcome, the malaria prevalence by PCR and anemia prevalence at 6 and 12 months after the implementation will be evaluated similarly. The cumulative malaria incidence during the 12-month follow-up will be analyzed based on the cohort follow-up data. Both symptomatic disease incidence and infection incidence, including asymptomatic infection, will be compared between the intervention and control arm based on cluster-level summaries. Entomological parameters, namely anopheline mosquito density, species composition, infection rate, and antibodies (IgG) against Anopheles salivary proteins gSG6 and cE5 at 6 and 12 months post-installation, will be analyzed in the same manner. Community members’ perceptions are analyzed qualitatively based on the questionnaire, focus group discussion, and in-depth interviews.

### Interim analyses {21b}

Since no interim analysis or stopping guidelines have been planned. From the aspect of the benefit of the population, a stepped wedge design will be followed after this trial.

### Methods for additional analyses (e.g., subgroup analyses) {20b}

No subgroup analyses are planned.

### Methods in analysis to handle protocol non-adherence and any statistical methods to handle missing data {20c}

Since the intervention (ceiling net) will be installed in trial participants’ houses, participants not sleeping in their own houses will not benefit from the intervention. In the cohort, non-adherence to the intervention can be inferred from travel history during bi-weekly interviews. Therefore, participants who regularly sleep outside their homes will be removed from the analyses. The extent and patterns of missing data will be assessed once all data collection has been completed. If necessary, multiple imputation methods will be used to handle missing data.

### Plans to give access to the full protocol, participant-level data, and statistical code {31c}

This manuscript is the full protocol. The corresponding author will make the de-identified datasets or any future statistical code available upon reasonable request.

### Oversight and monitoring

#### Composition of the coordinating center and trial steering committee {5d}

The sampling team, composed of CHVs and laboratory technicians, set up a day-to-day communication group and exchanged their experiences. A local management team of study investigators from Kenya and Japan also joined this, leading and advising the activities and monitoring the sample and data integrity. A monthly meeting will be held by the steering committee composed of all key researchers from Kenya and Japan, including the principal investigator (PI) and co-PI, which aim to monitor the progress of the trial.

#### Composition of the data monitoring committee, its role, and reporting structure {21a}

Because this intervention is considered to be of a low-risk nature, this study does not have a data monitoring committee. For additional credibility about study quality, the researchers will consult a third statistician, if necessary.

#### Adverse event reporting and harms {22}

Neither the ceiling net nor the synergist PBO is known to pose significant health or safety risks. Nonetheless, all unanticipated problems will be reported to the research team and Homa Bay County Ministry of Health (MOH) through CHVs. Medical officers from Homa Bay County will assess the relatedness of the reported events to the study and report to the research team, including the PI. In the event of a study-related serious adverse event, the study team will convene a meeting immediately with the MOH and Homa Bay County Teaching and Referral Hospital representatives to review the case and take necessary action.

#### Frequency and plans for auditing trial conduct {23}

After participant recruitment, enrollment, and implementation of the intervention are completed, the research team will have a meeting to review the protocols for outcome evaluation. A monthly meeting will be held during the follow-up period to ensure that all surveys and investigations are conducted according to the study protocol. The study is required to submit annual reports and renewal to ethical review boards of Osaka Metropolitan University, Japan, and Mount Kenya University, Kenya.

#### Plans for communicating important protocol amendments to relevant parties (e.g., trial participants, ethical committees) {25}

Decisions on important trial amendments must be made through a formal procedure and will be approved by institutional review boards (IRB) at Mount Kenya University and Osaka Metropolitan University. The protocol in the clinical trials registry will also be updated accordingly.

#### Dissemination plans {31a}

The results will be shared with the Homa Bay County government and Kenya National Malaria Control Program and discussed for the possibility of expanding the program. Also, the results will be disseminated through publications and conferences to help the development of novel malaria control strategies in other malaria-endemic countries. The feedback from the research participants will also help shape the future improvement of the intervention and acceptance by the communities.

## Discussion

Widespread adoption of LLIN has been cited as the most significant contributor to the decrease of malaria in sub-Saharan Africa between 2000 and 2015 [31]. However, insecticide resistance in *Anopheles* vectors, inadequate LLIN coverage, and non-compliance to LLIN hinder the effectiveness of LLIN. Olyset®Plus LLIN incorporates the synergist PBO to restore the insecticidal effect of permethrin against mosquitoes with metabolic resistance to the pyrethroid. By retrofitting Olyset®Plus LLIN into a ceiling net to protect those who are not covered by conventional LLIN, Olyset®Plus ceiling nets may represent an additional tool that can complement existing interventions to further reduce malaria transmission.

Ceiling nets have the potential to be more durable and sustainable than conventional LLIN. In our study area, many schoolchildren sleep on the floor [7], which makes the permanent installation of conventional LLIN difficult since it likely impedes movement within the house. Daily or frequent handling of LLIN may increase the risk of damage to it. Long-term observations from the previous ceiling net study [14] suggest that the overall physical integrity of the ceiling net is quite high even after 5 years, and even though the effect of the insecticide in the ceiling net is limited to 3 to 5 years [32], the physical protection provided by the ceiling net may still provide additional advantages over the use of conventional LLIN alone. Additional cost-effectiveness analyses and long-term observational studies on ceiling net durability will be useful to guide the adoption of this novel tool in the future.

As is typical of the wider Lake Victoria region of western Kenya, iron sheets are the most popular roofing material in our study area on Mfangano Island [23]. However, a variety of roof and house designs of various sizes makes the installation of ceiling nets a labor-intensive process. Using Olyset®Plus sheets 6 × 8 m or 4 × 6 m in size, it takes a team of three to four experienced installers approximately an hour to install the ceiling net in a typical house with a hip or tented roof. In this study, we provide training to CHVs and other village volunteers on ceiling net installations. If ceiling nets become more widely adopted in the future, an installation by experienced local volunteers or modification of the design should reduce the cost of implementation.

Communication with the community members is another key aspect of this trial. LLIN has been freely distributed to the communities every 3 years for more than a decade. Therefore, many residents may expect the same for the ceiling nets if the latter proves to be effective. Since ceiling nets are visible to all study participants, achieving adherence for 1 year, especially among participants in the control group, will be challenging. To encourage participant retention, a stepped wedge design that enables all households in the control arm to receive ceiling nets at the end of the 1-year follow-up period is planned after this trial. Since 2012, members of the research team have conducted collaborative community-based research projects with the local government and health authorities, stakeholders, and community gatekeepers in the study area [26, 33–35]. Good rapport with local community members is crucial to secure their participation in this study.

This study has several limitations. First, the visible nature of the ceiling net makes it impossible to blind study participants and field research staff. However, microscopists and laboratory technicians who process and analyze samples are blinded to the study arm assignment of participants. Indoor resting mosquitoes will be collected by pyrethrum spray catches and CDC light traps to determine vector density, which should be free of assessor bias. Second, since we hypothesize that schoolchildren will benefit the most from the additional protection of the ceiling net, our primary outcome is the prevalence of *Plasmodium* infections in children aged 3 to 15 years old. This will be assessed by school-based cross-sectional surveys. However, the benefit of the ceiling net will likely be extended to other age groups who share the same dwelling units. While we plan to examine the impact in a longitudinal cohort, the present cohort is small relative to the scale of the intervention. As such, findings in this cohort may not be generalizable to the trial population. Third, given that the ceiling nets are visible and thought to function in ways similar to conventional LLIN, it is conceivable that participants in the intervention arm may lower their usage of conventional LLIN, effectively relying on the ceiling net as a replacement rather than an addition to conventional LLIN for malaria protection. Continuous community engagement throughout the trial period will help to reinforce the importance of consistent LLIN use. Bi-weekly interviews with cohort participants will provide data on the pattern of LLIN use after ceiling net installation. We will conduct surveys and in-depth interviews to elicit participants’ perceptions of the ceiling net, which can guide future messaging and implementation.

Improving housing and the built environment, including screened windows, doors, or eaves to reduce malaria transmission, has long been proposed [15, 36]. However, there has been only a handful of trials on house modifications for preventing malaria [17], and only two have focused on the impact of screening of the ceiling on malaria prevalence [13, 14]. This study will be the first trial to evaluate the impact of ceiling nets on the infection incidence by active case detection, as submicroscopic or/and asymptomatic infections represent a known but not well-understood source of residual transmission. The results from this study will inform decision-makers about the wider adoption of ceiling nets as another tool in the next generation of vector control.

## Trial status

Recruitment started on September 1, 2021, and the final subject enrollment was completed on October 10, 2021. The follow-up of the cohort has been completed on December 16, 2022. The focus group discussion and questionnaire survey for the perception, as a final data collection, is planned for April 2023 (Table 3). The current protocol is version 2.1 as of June 22, 2021. The study was initially intended to be a stepped wedge trial; however, the authors agreed that the study should be published as a stand-alone cluster randomized controlled trial; thus, submission of this protocol was delayed.

**Table 3:** Trial status and plan

Action	Date
Anticipated date of first enrollment	September 1, 2021
The actual date of the first enrollment	September 23, 2021
Finalize the enrollment	October 10, 2021
Start the implementation of the intervention	November 22, 2021
Finalize all the implementation	January 21, 2022
Start the first sampling in the cohort	December 9, 2021
Complete all follow-ups in the cohort	December 16, 2022
Complete cross-sectional malaria survey	February 24, 2023
Qualitative data collection (completion of the data collection) (expected)	April 2023

## Data Availability

The study regimes, consent forms, assent forms, and study-related materials are accessible from the corresponding author. The final trial dataset will be available to all investigators. The corresponding author will make the de-identified datasets available upon reasonable request.

## Abbreviations

LLIN: Long-lasting insecticidal net
PBO: Piperonyl butoxide
ITN: Insecticide-treated net
RDT: Rapid diagnostic test
ACT: Artemisinin-based combination therapy
CHV: Community health volunteer
